# The Book World of Medicine and Science

**Published:** 1898-10-22

**Authors:** 


### The Book World of Medicine and Science.

Medical Diseases of Infancy and Childhood. By Dawson Williams, M.D. Lond., F.R.C.P., Physician to the East London Hospital for Children. (London: Cassell and Co. 1898. Pp. 634, with 36 Illustrations. Price 10s. 6d.)

This is a thoroughly good book—good as a complete and impartial presentment of the wide range of subjecs with which it deals, and good also as a clinical manual for the practitioner. The author is right in saying that pathological processes are essentially the same in children as in adults, but he has amply shown in the book before us how important it is to make a special study of the results of these pathological processes as they manifest themselves in those of tender age. After introductory chapters on the clinical examination of children, on the diseases incidental to birth, and on the all-important subject of food, the rest of the book is devoted to a consideration of the various diseases met with in child life, and finally there is a short appendix giving the doses of certain medicines, and a few prescriptions and recipes. We are told what is very true, that although in examining children it is of the utmost importance that every effort should be made to establish friendly relations with the patient, this is not the case with young infants; in fact, that it is, as a rule, waste of time to seek to conciliate them, or to divert their attention. This duty is better left to the nurse, and the examination should be methodically proceeded with, leaving to the last those things which are almost sure to cause crying, such as the examination of the fauces. The chapters on the acute specific infectious diseases are very full and complete, as, indeed, one would expect to be the case from the attention which the author is well known to have given to this subject. A little table which he introduces shows in a way which we think will be fresh to many of his readers the enormous preponderance of the infections as causes of infantile deaths. Taking a million infants under five years of age, 3,370 of them will die in a year from whooping-cough and 3,131 from measles; while of the same number of children between ten and fifteen years of age only four will die of whooping-cough and only twenty-three of measles. Dr. Dawson Williams well says then that the fact of the organism in infancy not having yet acquired immunity to the acute specific infectious diseases is one great cause of the difference between the pathological processes to which children are subject as compared with those which are met with among adults, for it is to these diseases that a very large part of the enormous mortality of the early years of life is attributable. The chapter on rickets is interesting, and the illustrations are very good. Dr. Dawson Williams attributes the deformity met with in the extremities of rickety children entirely to the effects of mechanical pressure. He points out that in the lower limbs the main deformity is in the bones of the legs, and that it is produced at the point where the legs cross each other as the child sits. "If, as is often the case, the child sits habitually with the same leg uppermost, the tibia of this leg is bowed inwards (upwards as the child sits)." It is to be noted that in the skiagram given of a case of knock-knee not only are the inner condyles of the femur elongated but the tibia and fibula are markedly bent. Under the head of diseases of the liver, we note that cirrhosis is attributed to alcoholism in only one-sixth of the cases. Next come syphilis and tuberculosis, and the major part of the rest are put down as sequelæ of acute infectious diseases, especially scarlet fever and measles. It is hardly fair, however, in reviewing a book to dip into it in this way, for where such care has been exercised, as has been done in the work before us, to deal impartially with the large number of subjects which are treated of, it would not be right to hold out any particular portion of the book as especially worthy of mention. Indeed, if one were to criticise anything in this book it would be its curious evenness and the self-denying way in which the author has kept himself in the background; all of which, although no doubt detracting somewhat from its literary style, tends to make the book all the more valuable as a work of reference to the practitioner in the ordinary everyday problems which he has to solve.

Atlas of Methods of Clinical Investigation, with an Epitome of Clinical Diagnosis and of Special Pathology and Treatment of Internal Diseases. By Christfried Jakob. Authorised translation from the German. Edited by Augustus A. Eshner, M.D. With 182 Coloured Illustrations and 64 Illustrations in the Text. (London: The Rebman Publishing Company. 1898. Price 12s. 6d. net.)

This is a careful, although somewhat free, translation of a book which has had considerable vogue in Germany, and is likely, we think, to be very acceptable to students in this country. They key-note of the book lies in its illustrations, the idea of the author being that a good pictorial presentment of any fact which bears such treatment is more likely to impress the mind than many words. Thus we find that the book commences with a series of plates representing the clinical microscopy and chemic colour-reactions of the blood and the various secretions and excretions. These plates are truly admirable, and form quite an atlas of clinical microscopy. Then come a series of diagrammatic representations of the disturbances of the various organs in disease, the normal projection of the viscera being first given, and then diagrams of the areas in which dulness on percussion and other altered physical signs are to be found in the different diseased conditions. These, although very good, and presenting the subject matter in a very striking manner, do not seem to us quite to repay the cost of the very elaborate plates on which they are given; that is to say, nearly all that is shown could have been shown, although not, perhaps, quite so clearly, by various stipplings in one colour. Still, as a means of impressing a fact on the memory the colour no doubt has its advantages. The only objection which we have to raise to this portion of the work is the crudity and harshness of the outlines, of the "dulness," and other physical signs, the edges of which in nature frequently fade off rather than ending suddenly as they do in the plates before us. When we come to the book itself we find a very careful and complete handbook of clinical diagnosis. We do not say that there is much in it which will not be met with in other works—in the larger text-books on medicine, for example—but the arrangement is very good and the matter is systematic, correct, and up-to-date. Finally, at the end there is a chapter giving some therapeutic notes on various methods of treatment and on some of the most important medicaments. Taken altogether, this is a very useful handbook for a student.

First Aid to the Injured. By Dr. Friedrich Esmarch. Translated from the German by H.R.H. Princess Christian. Sixth Edition. (London: Smith, Elder, and Co. 1898. Price 2s.)

A sixth edition of a book by so well-known a surgeon as Professor Esmarch, translated by a Princess so well known for her good works as H.R.H. Princess Christian, ought not to, and does not, require much notice from the reviewer. The book is intended to give to those who attend ambulance lectures a somewhat more detailed account of the work aimed at than is given in the official handbook, and it seems to fulfil its purpose very well. First, we have a sketch of the anatomy and physiology of the human body; then the various injuries and accidents to which it is subject are described; and, lastly, there are a couple of chapters, one on "Transport" and the other on "Nursing." We do not quite see the benefit which ambulance people are likely to gain from the description given of an operation for the removal of a tumour; otherwise the book seems likely to be useful.

